# Genetic susceptibility loci of idiopathic interstitial pneumonia do not represent risk for systemic sclerosis: a case control study in Caucasian patients

**DOI:** 10.1186/s13075-016-0923-3

**Published:** 2016-01-20

**Authors:** Minghua Wu, Shervin Assassi, Gloria A. Salazar, Claudia Pedroza, Olga Y. Gorlova, Wei V. Chen, Julio Charles, Miranda L. Taing, Kelley Liao, Fredrick M. Wigley, Laura K. Hummers, Ami A. Shah, Monique Hinchcliff, Dinesh Khanna, Elena Schiopu, Kristine Phillips, Daniel E. Furst, Virginia Steen, Murray Baron, Marie Hudson, Xiaodong Zhou, Janet Pope, Niall Jones, Peter Docherty, Nader A. Khalidi, David Robinson, Robert W. Simms, Richard M. Silver, Tracy M. Frech, Barri J. Fessler, Marvin J. Fritzler, Jerry A. Molitor, Barbara M. Segal, Malahat Movahedian, Javier Martín, John Varga, Maureen D. Mayes

**Affiliations:** Division of Rheumatology and Clinical Immunogenetics, Department of Internal Medicine, University of Texas McGovern Medical School at Houston, 6431 Fannin Street, Houston, TX 77030 USA; Department of Pediatrics, The University of Texas Mcgovern Medical School at Houston, 6431 Fannin Street, Houston, TX 77030 USA; Department of Community and Family Medicine, Geisel School of Medicine at Dartmouth, 1 Rope Ferry Road, Hanover, NH 03755 USA; Division of Rheumatology, Johns Hopkins University School of Medicine, 5501 Hopkins Bayview circle, Baltimore, MD 21224 USA; Division of Rheumatology, Northwestern University Feinberg School of Medicine, 240 East Huron Street, Chicago, IL 60611 USA; Department of Internal Medicine, Division of Rheumatology, University of Michigan Health Center, 300 North Ingalls Street, Ann Arbor, MI 48109 USA; Division of Rheumatology, University of California Los Angeles, 1000 Veterans Avenue, Los Angeles, CA 90024 USA; Division of Rheumatology, Georgetown University Medical Center, 3800 Reservoir Road, Washington, MD 20007 USA; Division of Rheumatology, McGill University, 1650 Cedar Avenue, Montreal, QC H3G 1A4 Canada; Division of Rheumatology, St. Joseph’s Health Care, University of Western Ontario, 268 Grosvenor Street, London, ON Canada; Division of Rheumatology, University of Alberta, 11405-87 Avenue, Edmonton, AB T6G 1C9 Canada; Division of Rheumatology, Moncton Hospital, 135 MacBeath Avenue, Moncton, NB E1C 6Z8 Canada; Division of Rheumatology, McMaster University at Hamilton, 1280 Main Street West, Hamilton, ON L8S 4L8 Canada; Division of Rheumatology, University of Manitoba, 820 Sherbrook Street, Winnipeg, MB R3A 1R9 Canada; Division of Rheumatology, Boston University School of Medicine, 72 East Concord Street, Boston, MA 02118 USA; Division of Rheumatology, Medical University of South Carolina, 171 Ashley Avenue, Charleston, SC 29425 USA; Division of Rheumatology, University of Utah, 30 North 1900 East, Salt Lake City, UT 84132 USA; Division of Clinical Immunology and Rheumatology, University of Alabama at Birmingham, 1825 University Boulevard, Birmingham, AB 35294 USA; Division of Rheumatology, University of Calgary Cumming School of Medicine, 3330 Hospital Drive NW, Calgary, AB T2N 4N1 Canada; Division of Rheumatology, University of Minnesota, 420 Delaware Street SE, Minneapolis, MN 55455 USA; Instituto de Parasitología y Biomedicina López-Neyra, Consejo Superior de Investigaciones Cientıficas, C/Ventanilla 11, 18001 Granada, Spain

**Keywords:** Idiopathic interstitial pneumonia (IIP), SSc-ILD, Genetic susceptibility

## Abstract

**Background:**

Systemic sclerosis (SSc)-related interstitial lung disease (ILD) has phenotypic similarities to lung involvement in idiopathic interstitial pneumonia (IIP). We aimed to assess whether genetic susceptibility loci recently identified in the large IIP genome-wide association studies (GWASs) were also risk loci for SSc overall or severity of ILD in SSc.

**Methods:**

A total of 2571 SSc patients and 4500 healthy controls were investigated from the US discovery GWAS and additional US replication cohorts. Thirteen IIP-related selected single nucleotide polymorphisms (SNPs) were genotyped and analyzed for their association with SSc.

**Results:**

We found an association of SSc with the SNP rs6793295 in the *LRRC34* gene (OR = 1.14, CI 95 % 1.03 to 1.25, *p* value = 0.009) and rs11191865 in the *OBFC1* gene (OR = 1.09, CI 95 % 1.00 to 1.19, *p* value = 0.043) in the discovery cohort. Additionally, rs7934606 in *MUC2* (OR = 1.24, CI 95 % 1.01 to 1.52, *p* value = 0.037) was associated with SSc-ILD defined by imaging. However, these associations failed to replicate in the validation cohort. Furthermore, SNPs rs2076295 in *DSP* (β = -2.29, CI 95 % -3.85 to -0.74, *p* value = 0.004) rs17690703 in *SPPL2C* (β = 2.04, CI 95 % 0.21 to 3.88, *p* value = 0.029) and rs1981997 in *MAPT* (β = 2.26, CI 95 % 0.35 to 4.17, *p* value = 0.02) were associated with percent predicted forced vital capacity (FVC%) even after adjusting for the anti-topoisomerase (ATA)-positive subset. However, these associations also did not replicate in the validation cohort.

**Conclusions:**

Our results add new evidence that SSc and SSc-related ILD are genetically distinct from IIP, although they share phenotypic similarities.

**Electronic supplementary material:**

The online version of this article (doi:10.1186/s13075-016-0923-3) contains supplementary material, which is available to authorized users.

## Background

Systemic sclerosis or scleroderma (SSc) is a complex autoimmune disease characterized by vasculopathy, autoantibody production and fibrosis in the skin and internal organs. The etiology of SSc remains unknown; effective treatments that target the underlying pathophysiology of SSc are unavailable and the disease-related mortality remains high [1]. Specifically, pulmonary fibrosis and pulmonary arterial hypertension (PAH) account for the majority of disease-related deaths in SSc [2]. SSc-associated interstitial lung disease (SSc-ILD) is usually characterized by a histologic pattern of nonspecific interstitial pneumonia (NSIP) or, less frequently, usual interstitial pneumonia (UIP). SSc-ILD has clinical and radiologic similarities to idiopathic interstitial pneumonia (IIP). A polymorphism in the MUC5B promoter region was strongly associated with familial and idiopathic pulmonary fibrosis (IPF; the most common IIP type) [3], but it has not been found to be a susceptibility locus for SSc or SSc-associated interstitial lung disease (SSc-ILD) [4–6].

Recently, large genome-wide association studies (GWASs) in fibrotic IIP (n = 1616 patients) and IPF (n = 542 patients and two validation cohorts n = 544 and n = 324) identified/confirmed susceptibility single nucleotide polymorphisms (SNPs) in *TERT*, *AZGP1*, *MUC2*, *IVD*, *DSP*, *MAPT*, *DPP9*, *LRRC34*, *FAM13A*, *OBFC1*, *CSMD1*, *ATP11A* [7] and *TOLLIP*, *MDGA2*, *SPPL2C* [8]. Given the clinical and radiologic similarities between SSc-ILD and IIP, we examined the association of the 13 SNPs among the listed genes, identified in the above IIP and IPF GWASs [7, 8] (*TOLLIP* and *MDGA2* genes were excluded because the related SNPs were not present on the Illumina BeadChip utilized in SSc GWAS) with SSc as a single disease entity, with SSc-ILD by imaging, or SSc-ILD severity (as determined by percent predicted forced vital capacity (FVC%)) in two large SSc patient samples.

## Methods

### Study population

Two non-Hispanic white populations (discovery and replication cohorts) were investigated. For the discovery cohort, we utilized data from our previously published SSc GWAS study consisting of 1486 SSc cases (patients from the US) and 3477 unaffected race- and ethnicity-matched controls [9]. Selected polymorphisms were genotyped in an independent replication cohort consisting of 1085 additional SSc cases (patients from the US and Canada) and 1023 additional unaffected controls. Patients were recruited at the University of Texas – Houston and from the following sites: the participating Canadian Scleroderma Research Group (CSRG) sites, University of California Los Angeles, University of Michigan, Georgetown University, Boston University, Medical University of South Carolina, Johns Hopkins University, University of Utah, Northwestern University, University of Alabama at Birmingham and University of Minnesota. All patients were enrolled in the National Scleroderma Family Registry and DNA Repository. All patients with SSc fulfilled the 1980 American College of Rheumatology classification criteria for SSc or had at least three of the five CREST (Calcinosis, Raynaud’s phenomenon, Esophageal dysmotility, Sclerodactyly, Telangiectasias) features (Table 1).

**Table 1:** Demographic and clinical features of SSc patients in the discovery and replication cohorts

	Discovery (n = 1486)	Replication (n = 1085)
Female	1307 (88.0 %)	934 (86.1 %)
Mean age ± years	54.5 ± 12.9 y	56.1 ± 12.8 y
Diffuse SSc	505 (34.0 %)	403 (37.1 %)
Disease duration	10.1 ± 8.7 y	10.9 ± 9.6 y
ACA	425 (28.6 %)	340 (31.3 %)
ATA	238 (16.0 %)	180 (16.6 %)
SSc-ILD		
Yes	185 (65.6 %)	313 (61.9 %)
No	97 (34.4 %)	193 (38.1 %)

*ACA* anti-centromere antibody, *ATA* anti-topoisomerase 1 antibody, *SSc* systemic sclerosis, *ILD* interstitial lung disease

The genotypes of unaffected controls for the discovery cohort were obtained from the Cancer Genetic Markers of Susceptibility (CGEMS; non-cancer healthy controls) studies and Illumina iControlDB database (www.illumina.com/iControlDB, Illumina, San Diego, CA, USA). The unaffected controls for the replication cohort were recruited through a nationwide effort by the Scleroderma Family Registry and DNA Repository.

Collection of blood samples and clinical information from case and control subjects was undertaken with fully informed consent and relevant ethical review board approval from each contributing center in accordance with the tenets of the Declaration of Helsinki.

### SNP selection and genotyping assay

In the discovery cohort, genotyping was performed using the Illumina Bead-Array GWAS platform. Specifically, patients were genotyped using Illumina Human 610-Quad BeadChip and controls were genotyped on Illumina Hap550K-BeadChip [9]. The 13 single nucleotide polymorphisms (SNPs) identified/confirmed to be associated with IIP [7], rs2736100 (*TERT*), rs2076295 (*DSP*), rs4727443 (*AZGP1*), rs7934606 (*MUC2*), rs2034650 (*IVD*), rs1981997 (*MAPT*), rs12610495 (*DPP9*), rs6793295 (*LRRC34*), rs2609255 (*FAM13A*), rs11191865 (*OBFC1*), rs1278769 (*ATP11A*), rs1379326 (*CSMD1*) and an additional SNP rs17690703 (*SPPL2C*) [8], were investigated in the discovery cohort. We also investigated association of 13 SNPs with anti-topoisomerase 1 antibody (ATA) and anti-centromere antibody (ACA) positive subgroup patients. The SNPs reaching a nominal level of significance (*p* < 0.05) in the discovery cohort (rs6793295 and rs11191865) were genotyped in the replication cohort using TaqMan allele discrimination assays in a 7900HT fast real-time PCR system (Applied Biosystems, Foster City, CA, USA).

### SSc-ILD and severity of SSc-ILD

We also investigated the association of the above SNPs with SSc-ILD by imaging. We compared the frequency of the above SNPs in patients with SSc-ILD to unaffected controls. SSc-ILD was defined by chest high-resolution computed tomography (HRCT)/chest X-ray images. SSc patients (total = 498; n = 185 in the discovery cohort and n = 313 in the replication cohort) were considered to have interstitial lung disease (ILD) by imaging if they had: (1) ground glass opacity or increased interstitial markings on chest HRCT; or (2) increased basilar reticular marking on chest X-ray (in total, 498 patients, 185 in the discovery cohort and 313 in the replication cohort had ILD). The number of patients with imaging-proven ILD is low because HRCT imaging results were available only n = 212 in the discovery cohort and n = 347 in the replication cohort patients. The case-case comparison in regard to presence of ILD was compared by the low number of patients with available HRCT imaging in the discovery cohort.

Furthermore, an association with severity of ILD was investigated. Percent predicted forced vital capacity (FVC%) (measured in a total of 1954 patients; n = 1072 in the discovery cohort and n = 882 in the replication cohort) as a continuous variable was used as a surrogate for severity of SSc-ILD as this has been demonstrated to be a validated outcome measure for severity of ILD in randomized controlled studies of patients with SSc [10]. SNPs reaching the nominal significance level (*p* < 0.05) in these two analyses were also genotyped in the replication cohort (rs7934606, rs2076295, rs17690703 and rs1981997).

### Statistical analysis

We followed the same genetic inheritance modes for each specific SNP that was utilized in the IIP GWAS [7]. Specifically, all SNPs except for rs1379326 (*CSMD1*) were investigated in an additive model. Similar to the IIP GWAS [7], rs1379326 (*CSMD1*) was investigated in a recessive model. The additive model corresponds to the risk or protective effect conferred by the rarer (minor) allele (0, 1 or 2 copies) as a predictor of phenotypic status; and the recessive model corresponds to the effect conferred by only homozygous status for the rarer allele. Genotype data quality was verified for each SNP by testing for Hardy-Weinberg equilibrium (HWE). Hardy-Weinberg equilibrium was assessed by an *x*^*2*^ test or Fisher’s exact test. None of the included cohorts showed significant deviation from HWE for all the genotyped SNPs. Logistic regression that included both patients and controls was utilized to examine the association of the above SNPs with SSc overall and SSc-ILD. A linear regression model was used to investigate the association of these genetic variants with FVC% predicted (data was normally distributed) as a continuous variable, among patients only. FVC% predicted was investigated as a continuous rather than a dichotomous variable to increase our power to detect a difference in FVC levels conditional on the genotype. Anti-topoisomerase 1 antibody (ATA) status was included as a potential confounder in the multivariable regression model. The combined analysis of the discovery and the replication cohorts was performed via a random-effects meta-analysis model.

## Results

### Discovery cohort

We first investigated whether the 13 IIP-associated polymorphisms were associated with risk of SSc overall or with SSc-ILD. Several SNPs showed nominally significant associations with SSc in the discovery cohort (Table 2). Specifically, *LRRC34* rs6793295 (OR = 1.14, CI 95 % 1.03 to 1.25, *p* value = 0.009) and *OBFC1* rs11191865 (OR = 1.09, CI 95 % 1.00 to 1.19, *p* value = 0.043) were associated with SSc compared to controls (Table 2). There were no significant associations observed when the SNP frequencies in SSc antibody subgroups (ATA- or ACA-positive patients) were compared to controls (Table 3).

**Table 2:** Association between the investigated genotypes in SSc-versus-control comparisons in the discovery cohort

SNP	Gene	Minor allele^*^	MAF (case)^**^	OR (95 % CI)	*p* value
rs2736100	*TERT*	T	0.50	1.04 (0.95; 1.13)^†^	0.393
rs2076295	*DSP*	G	0.44	0.96 (0.88; 1.05)^†^	0.387
rs4727443	*AZGP1*	A	0.41	1.03 (0.95; 1.13)^†^	0.487
rs7934606	*MUC2*	A	0.41	1.08 (0.99; 1.18)^†^	0.084
rs2034650	*IVD*	G	0.49	1.03 (0.95; 1.13)^†^	0.458
rs1981997	*MAPT*	A	0.22	0.97 (0.88; 1.08)^†^	0.636
rs12610495	*DPP9*	G	0.28	1.00 (0.91; 1.10)^†^	0.967
**rs6793295**	***LRRC34***	**C**	**0.29**	**1.14 (1.03; 1.25)** ^†^	**0.009**
rs2609255	*FAM13A*	G	0.22	1.01 (0.91; 1.12)^†^	0.851
**rs11191865**	***OBFC1***	**G**	**0.52**	**1.09 (1.00; 1.19)** ^†^	**0.043**
rs1278769	*ATP11A*	A	0.24	0.95 (0.86; 1.06)^†^	0.365
rs1379326	*CSMD1*	G	0.26	0.86 (0.69; 1.08)^‡^	0.199
rs17690703	*SPPL2C*	T	0.26	1.03 (0.95; 1.11)	0.546

Gene names: *TERT*: telomerase reverse transcriptase; *DSP*: desmoplakin; *AZGP1*: alpha-2-glycoprotein 1, zinc-binding; *MUC2*: mucin 2; *IVD*: isovaleryl-CoA dehydrogenase; *MAPT*: microtubule-associated protein tau; *DPP9*: dipeptidyl-peptidase 9; *LRRC34*: leucine-rich repeat-containing 34; *FAMI3A*: family with sequence similarity 13, member A; *OBFC1*: oligonucleotide/oligosaccharide-binding fold containing 1; *ATP11A*: ATPase, class VI, type 11A; *CSMD1*: CUB and Sushi multiple domains 1; *SPPL2C*: signal peptide peptidase-like 2C

*SSc* systemic sclerosis, *SNP* single nucleotide polymorphism, *MAF* minor allele frequency

^*^The minor allele is defined as the minor allele in the combined case and control group; ^**^MAF is defined as the minor allele frequency in cases and controls; ^†^Odds ratio for the minor allele in the additive model; ^‡^Odds ratio based on the recessive model

**Table 3:** Association studies of investigated genotypes in comparison of ATA- or ACA-positive SSc patients to controls in the discovery cohort

		ATA (+)		ACA (+)	
SNP	Gene	OR (95 % CI)	*p* value	OR (95 % CI)	*p* value
rs2736100^*^	*TERT*	1.01 (0.84; 1.22)	0.881	1.14 (0.99; 1.31)	0.069
rs2076295^*^	*DSP*	1.01 (0.83; 1.21)	0.945	0.94 (0.82; 1.09)	0.423
rs4727443^*^	*AZGP1*	1.12 (0.93; 1.36)	0.226	0.96 (0.83; 1.11)	0.609
rs7934606^*^	*MUC2*	1.00 (0.83; 1.20)	0.992	1.07 (0.93; 1.23)	0.925
rs2034650^*^	*IVD*	0.99 (0.82; 1.19)	0.915	1.04 (0.90; 1.20)	0.584
rs1981997^*^	*MAPT*	0.92 (0.73; 1.16)	0.495	1.15 (0.97; 1.36)	0.100
rs12610495^*^	*DPP9*	0.98 (0.80; 1.20)	0.847	1.05 (0.90; 1.22)	0.572
rs6793295^*^	*LRRC34*	1.15 (0.94; 1.42)	0.172	1.15 (0.98; 1.35)	0.080
rs2609255^*^	*FAMI3A*	1.04 (0.83; 1.30)	0.736	1.00 (0.84; 1.19)	1.000
**rs11191865** ^*^	***OBFC1***	**1.21 (1.00; 1.45)**	**0.048**	0.99 (0.86; 1.14)	0.898
rs1278769^*^	*ATP11A*	1.09 (0.89; 1.35)	0.394	0.96 (0.81; 1.14)	0.650
rs1379326^†^	*CSMD1*	0.78 (0.46; 1.31)	0.340	0.94 (0.65; 1.36)	0.736
rs17690703	*SPPL2C*	0.97 (0.76; 1.26)	0.870	1.14 (0.94; 1.38)	0.184

Gene names: *TERT*: telomerase reverse transcriptase; *DSP*: desmoplakin; *AZGP1*: alpha-2-glycoprotein 1, zinc-binding; *MUC2*: mucin 2; *IVD*: isovaleryl-CoA dehydrogenase; *MAPT*: microtubule-associated protein tau; *DPP9*: dipeptidyl-peptidase 9; *LRRC34*: leucine-rich repeat-containing 34; *FAMI3A*: family with sequence similarity 13, member A; *OBFC1*: oligonucleotide/oligosaccharide-binding fold containing 1; *ATP11A*: ATPase, class VI, type 11A; *CSMD1*: CUB and Sushi multiple domains 1; *SPPL2C*: signal peptide peptidase-like 2C

*ATA* anti-topoisomerase 1 antibody, *ACA* anti-centromere antibody, *SSc* systemic sclerosis, *SNP* single nucleotide polymorphism

^*^Additive model;,^†^Recessive model

Next, we investigated the association in the discovery cohort between the above polymorphisms and SSc-ILD by imaging or FVC% predicted in order to investigate the relationship with SSc-ILD presence or severity. We also performed case-case comparison by presence of ILD with no ILD in SSc. Interestingly, rs7934606 in *MUC2* (OR = 1.24, CI 95 % 1.01 to 1.52, *p* value = 0.037) was associated with SSc-ILD by imaging when compared to controls (Table 4). Furthermore, three polymorphisms *DSP* rs2076295 (β = -2.29, CI 95 % -3.85 to -0.74, *p* value = 0.004) *SPPL2C* rs17690703 (β = 2.04, CI 95 % 0.21 to 3.88, *p* value = 0.029) and *MAPT* rs1981997 (β = 2.26, CI 95 % 0.35 to 4.17, *p* value = 0.02) were associated with FVC% predicted (Table 5). Of note, the minor allele (A) of *MAPT* rs1981997 and (T) of *SPPL2C* rs17690703 were associated with higher FVC% predicted, which is consistent with the IIP GWAS results with the minor allele being protective in that study (OR < 1) [7]. Even after adjustment for ATA status, the above three polymorphisms showed nominally significant associations with ILD severity in SSc patients (Table 5). *SPPL2C* rs17690703 was associated ILD-SSc compared to SSc with no ILD as determined by imaging in the discovery cohort (Table S1 in Additional file 1).

**Table 4:** Association between the investigated genotypes in SSc-ILD (by imaging) patients compared to controls in the discovery and replication cohort

		SSc-ILD vs control (discovery cohort)		SSc-ILD vs control (replication cohort)	
SNP (coded allele)	Gene	OR (95 % CI)	*p* value	OR (95 % CI)	*p* value
rs2736100 (T)^*^	*TERT*	1.14 (0.93; 1.40)	0.199		
rs2076295 (G)^*^	*DSP*	1.13 (0.92; 1.39)	0.243		
rs4727443 (A)^*^	*AZGP1*	0.89 (0.71; 1.10)	0.270		
**rs7934606 (A)** ^*^	***MUC2***	**1.24 (1.01; 1.52)**	**0.037**	1.14 (0.81; 1.63)	0.444
rs2034650 (C)^*^	*IVD*	0.86 (0.70; 1.06)	0.152		
rs1981997 (A)^*^	*MAPT*	0.81 (0.62; 1.06)	0.123		
rs12610495 (G)^*^	*DPP9*	1.17 (0.93; 1.46)	0.179		
rs6793295 (C)^*^	*LRRC34*	1.02 (0.80; 1.30)	0.863		
rs2609255 (G)^*^	*FAMI3A*	0.99 (0.77; 1.27)	0.919		
rs11191865 (G)^*^	*OBFC1*	1.08 (0.87; 1.32)	0.496		
rs1278769 (A)^*^	*ATP11A*	1.00 (0.79; 1.28)	0.987		
rs1379326 (G)^†^	*CSMD1*	0.68 (0.37; 1.27)	0.224		
rs17690703 (T)^*^	*SPPL2C*	1.23 (0.87; 1.72)	0.238		

Gene names: *TERT*: telomerase reverse transcriptase; *DSP*: desmoplakin; *AZGP1*: alpha-2-glycoprotein 1, zinc-binding; *MUC2*: mucin 2; *IVD*: isovaleryl-CoA dehydrogenase; *MAPT*: microtubule-associated protein tau; *DPP9*: dipeptidyl-peptidase 9; *LRRC34*: leucine-rich repeat-containing 34; *FAMI3A*: family with sequence similarity 13, member A; *OBFC1*: oligonucleotide/oligosaccharide-binding fold containing 1; *ATP11A*: ATPase, class VI, type 11A; *CSMD1*: CUB and Sushi multiple domains 1; *SPPL2C*: signal peptide peptidase-like 2C

*SSc* systemic sclerosis, *ILD* interstitial lung disease, *SNP* single nucleotide polymorphism

^*^Additive model; ^†^Recessive model

**Table 5:** Association between the investigated genotypes and FVC% predicted in SSc patients in the discovery cohort

		FVC%		FVC% (adjusted for ATA status)	
SNP (Coded allele)	Gene	β (95 % CI)	*p* value	β (95 % CI)	*p* value
rs2736100 (T)^*^	*TERT*	0.51 (-1.04; 2.05)	0.521	0.76 (-0.81; 2.34)	0.343
**rs2076295 (G)** ^*^	***DSP***	**−2.29 (-3.85; -0.74)**	**0.004**	**−2.52 (-4.09; -0.95)**	**0.002**
rs4727443 (A)^*^	*AZGP1*	−0.27 (-1.85; 1.32)	0.742	0.08 (-1.53; 1.70)	0.919
rs7934606 (A)^*^	*MUC2*	−0.55 (-2.16; 1.05)	0.499	−0.75 (-2.37; 0.86)	0.360
rs2034650 (C)^*^	*IVD*	0.67 (-0.90; 2.25)	0.402	0.37 (-1.22; 1.97)	0.646
**rs1981997 (A)** ^*^	***MAPT***	**2.26 (0.35; 4.17)**	**0.020**	**3.19 (1.23; 5.14)**	**0.001**
rs12610495 (G)^*^	*DPP9*	0.57 (-1.18; 2.32)	0.523	1.05 (-0.72; 2.82)	0.246
rs6793295 (C)^*^	*LRRC34*	−0.46 (-2.19;1.27)	0.599	−1.05 (-2.82; 0.71)	0.242
rs2609255 (G)^*^	*FAMI3A*	1.72 (-0.10; 3.55)	0.064	1.61 (-0.18; 3.41)	0.079
rs11191865 (G)^*^	*OBFC1*	−0.73 (-2.27; 0.82)	0.356	0.02 (-1.55; 1.60)	0.977
rs1278769 (A)^*^	*ATP11A*	−0.62 (-2.45; 1.20)	0.503	−0.74 (-2.60; 1.11)	0.433
rs1379326 (G)^†^	*CSMD1*	3.35 (-0.84; 7.54)	0.117	3.12 (-1.08; 7.31)	0.145
**rs17690703 (T)** ^*^	***SPPL2C***	**2.04 (0.21; 3.88)**	**0.029**	**2.11 (0.30; 3.91)**	**0.022**

Gene names: *TERT*: telomerase reverse transcriptase; *DSP*: desmoplakin; *AZGP1*: alpha-2-glycoprotein 1, zinc-binding; *MUC2*: mucin 2; *IVD*: isovaleryl-CoA dehydrogenase; *MAPT*: microtubule-associated protein tau; *DPP9*: dipeptidyl-peptidase 9; *LRRC34*: leucine-rich repeat-containing 34; *FAMI3A*: family with sequence similarity 13, member A; *OBFC1*: oligonucleotide/oligosaccharide-binding fold containing 1; *ATP11A*: ATPase, class VI, type 11A; *CSMD1*: CUB and Sushi multiple domains 1; *SPPL2C*: signal peptide peptidase-like 2C

*FVC%* percent predicted forced vital capacity, *SSc* systemic sclerosis, *ATA* anti-topoisomerase 1 antibody, *SNP* single nucleotide polymorphism

^*^Additive model; ^†^Recessive model

### Replication cohort and combined analysis

We genotyped the samples from the validation set for the above six SNPs, which either showed nominally significant associations (*p* < 0.05) with risk of SSc overall (*LRRC34* rs6793295 and *OBFC1* rs11191865) or with SSc-ILD (rs7934606 in *MUC2*) and FVC% predicted (*DSP* rs2076295, *SPPL2C* rs17690703 and *MAPT* rs1981997). However, these associations were not found in the replication cohort (Table 4 and upper part of Table 6). Contrary to the discovery cohort, *MAPT* rs1981997 (OR = 0.79; CI 95 % 0.68 to 0.91; *p* value = 0.002) and *SPPL2C* rs17690703 (OR = 0.64; CI 95 % 0.49 to 0.83; *p* value = 0.001) were associated with SSc susceptibility but not with ILD severity in the replication cohort (Table 6). Furthermore, *SPPL2C* rs17690703 was not associated with SSc-ILD when compared to SSc without ILD by imaging in the validation cohort (Table S1 in Additional file 1).

**Table 6:** Association analysis of investigated genotypes in SSc versus control comparison as well as FVC% in the replication and combined cohorts

			Allele frequency		FVC%	FVC%–adjusted for ATA		
	SNPs	Gene	OR (95 % CI)	*p* value	β (95 % CI)	*p* value	β (95 % CI)	*p* value
Replication	rs2076295 (G)^†^	*DSP*	0.93 (0.82; 1.05)	0.252	0.32 (-1.49; 2.13)	0.729	0.39 (-1.40; 2.17)	0.671
	**rs1981997 (A)** ^†^	***MAPT***	**0.79 (0.68; 0.91)**	**0.002**	0.15 (-2.05; 2.35)	0.894	−0.01 (-2.17; 2.15)	0.994
	rs6793295 (C)^†^	*LRRC34*	1.06 (0.92; 1.21)	0.435	−0.54 (-2.53;1.45)	0.593	−0.55 (-2.51; 1.41)	0.581
	rs11191865 (G)^†^	*OBFC1*	0.90 (0.80; 1.01)	0.093	−0.34 (-2.11; 1.44)	0.710	−0.30 (-2.05; 1.45)	0.736
	**rs17690703 (T)** ^†^	***SPPL2C***	**0.64 (0.49; 0.83)**	**0.001**	2.20 (-1.38; 5.78)	0.227	3.11 (-0.45; 6.66)	0.086
Combined	rs2076295 (G)^†^	*DSP*	0.95 (0.89; 1.02)	0.171	−1.02 (-3.58; 1.54)	0.579	−1.07 (-3.94; 1.80)	0.079
	rs1981997 (A)^†^	*MAPT*	0.89 (0.78; 1.02)	0.091	1.26 (-0.81; 3.32)	0.445	1.59 (-1.56; 4.74)	0.503
	**rs6793295 (C)** ^†^	***LRRC34***	**1.11 (1.03; 1.20)**	**0.010**	−0.50 (-1.80; 0.81)	0.590	−0.81 (-2.12; 0.50)	0.440
	rs11191865 (G)^†^	*OBFC1*	1.01 (0.90; 1.13)	0.912	−0.55 (-1.72; 0.61)	0.524	−0.13 (-1.30; 1.04)	0.868
	**rs17690703 (T)** ^†^	***SPPL2C***	1.04 (0.97; 1.13)	0.221	**2.32 (0.68; 3.96)**	**0.006**	−1.03 (-4.67; 2.60)	0.575

Gene names: *TERT*: telomerase reverse transcriptase; *DSP*: desmoplakin; *AZGP1*: alpha-2-glycoprotein 1, zinc-binding; *MUC2*: mucin 2; *IVD*: isovaleryl-CoA dehydrogenase; *MAPT*: microtubule-associated protein tau; *DPP9*: dipeptidyl-peptidase 9; *LRRC34*: leucine-rich repeat-containing 34; *FAMI3A*: family with sequence similarity 13, member A; *OBFC1*: oligonucleotide/oligosaccharide-binding fold containing 1; *ATP11A*: ATPase, class VI, type 11A; *CSMD1*: CUB and Sushi multiple domains 1; *SPPL2C*: signal peptide peptidase-like 2C

*SSc* systemic sclerosis, *FVC %* percent predicted forced vital capacity, *ATA* anti-topoisomerase 1 antibody, *SNP* single nucleotide polymorphism

^†^Additive model

In the combined discovery and replication cohorts (lower part of Table 6), only the *LRRC34* rs6793295 was associated with risk of SSc overall (OR = 1.11; CI 95 % 1.03 to 1.2; *p* value = 0.01). However, the observed significance level was not stronger in the combined cohort than in the discovery cohort and this association did not withstand correction for multiple comparisons. Specifically, the *p* value = 0.13 in the combined cohort after Bonferroni correction for multiple comparisons. Furthermore, *LRRC34* rs6793295 was not associated with FVC% predicted in the combined cohort. *SPPL2C* rs17690703 was associated with FVC% predicted in the combined cohort but not after ATA adjustment or with overall SSc risk (Table 6). The observed association of *SPPL2C* rs17690703 with FVC% did not withstand correction for multiple comparison (*p*_corr_ = 0.078).

## Discussion

The aim of this study was to assess genetic components of SSc and SSc-related ILD, specifically to determine if SSc-ILD and IIP share common genetic risk factors. We analyzed 13 SNPs which showed robust association with IIP in a previous GWAS report [7, 8]. However, we failed to replicate these associations with SSc or SSc-related ILD in a large North American cohort.

Interstitial lung disease (ILD) is the most common pulmonary manifestation in patients with SSc [11] and is the most frequent cause of SSc disease-related death [12]. ILD occurs with greater frequency and increased severity in patients with diffuse cutaneous SSc (dc-SSc) and in ATA-positive patients. In SSc-ILD, loss of lung function and extent of fibrosis are the most important prognostic factors. It is known that genetic factors contribute not only to SSc susceptibility, but also to predisposition to SSc clinical phenotypes including disease type (limited versus diffuse cutaneous disease) and autoantibody status [13]. Recently, several lines of evidence have suggested that, even though SSc-ILD has phenotypic similarities to IIP, the genetic risk factors for these two conditions are quite different. For example the MUC5B promoter region polymorphism (rs868903), which was strongly associated with familial and idiopathic pulmonary fibrosis [3, 7], was not identified as a susceptibility locus for SSc or SSc–associated interstitial lung disease [4–6]. In addition, the two large SSc GWAS reports (each with independent discovery and validation cohorts) identified the major histocompatibility complex (MHC) class II region as being the most strongly associated region with SSc [9, 14], whereas MHC loci were not found to be risk factors in the IIP GWAS [7].

The present study has some limitations. In our study, only a subgroup of SSc patients had undergone HRCT. We performed a case-case comparison in regard to presence of SSc-ILD versus no SSc-ILD in the low number of patients because HRCT results were not available in the remainder of patients. The genotype results for *TOLLIP* and *MDGA2* genes were not available on our GWAS platform and could not be investigated. Furthermore, this is a cross-sectional study, thus, we cannot examine whether the investigated genetic loci have predictive significance for ILD progression. However, mean disease duration of this study was more than 10 years and most of the SSc-ILD cases had been already established. Therefore, we believe the cross-sectional FVC% is a reasonable surrogate for severity of ILD.

## Conclusions

In this study, we confirm that genetic susceptibility loci for IIP are not risk loci for SSc or severity of SSc-ILD in two validation SSc cohorts of non-Hispanic white ethnic background. Our findings and those of previous genetic susceptibility studies in SSc [15] implicate pathways in innate and adaptive immunity, whereas susceptibility loci for IIP relate to epithelial cell injury/dysfunction and abnormal wound healing [16]. Future challenges will be to identify the functional relevance of these variants for the final common pathway that results in the phenotype of fibrotic lung disease. This study represents an important step forward toward a better understanding of the complex genetic association of SSc particularly with lung involvement. We add new evidence that SSc and SSc-ILD are genetically distinct from IIP.

## Additional file

Additional file 1: Table S1. Association between the investigated genotypes in SSc-ILD (by imaging) patients compared to SSc-no ILD (by imaging) in the discovery and replication cohort. (DOCX 19 kb)

## Abbreviations

ACA: Anti-centromere antibody
ATA: Anti-topoisomerase 1 antibody
CGEMS: Cancer Genetic Markers of Susceptibility
CREST: Calcinosis, Raynaud’s phenomenon, Esophageal dysmotility, Sclerodactyly, Telangiectasias
dcSSc: Diffuse cutaneous SSc
FVC%: Percent predicted forced vital capacity
GWAS: Genome-wide association study
HRCT: High-resolution computed tomography
HWE: Hardy-Weinberg equilibrium
IIP: Idiopathic interstitial pneumonia
ILD: Interstitial lung disease
IPF: Idiopathic pulmonary fibrosis
MHC: Major histocompatibility complex
NSIP: Nonspecific interstitial pneumonia
PAH: Pulmonary arterial hypertension
SNP: Single nucleotide polymorphisms
SSc: Systemic sclerosis
SSc-ILD: SSc-associated interstitial lung disease
UIP: Usual interstitial pneumonia
