# Supplementary material for: Genetic susceptibility loci of idiopathic interstitial pneumonia do not represent risk for systemic sclerosis: a case control study in Caucasian patients
Source: Arthritis Res Ther. 2016 Jan 20;18:20. doi: 10.1186/s13075-016-0923-3 (PMC4719560; doi:10.1186/s13075-016-0923-3)
Supplement: Additional file 1: Table S1. — Association between the investigated genotypes in SSc-ILD (by imaging) patients compared to SSc-no ILD (by imaging) in the discovery and replication cohort. (DOCX 19 kb) [file 13075_2016_923_MOESM1_ESM.docx]

**Supplemental Table 1. Association between the investigated genotypes in SSc-ILD (by imaging) patients compared to SSc-no ILD (by imaging) in the discovery and replication cohort**

|  |  | SSc-ILD Vs SSc-no ILD (Discovery cohort) | | | SSc-ILD Vs no ILD (Replication cohort) | | |
| --- | --- | --- | --- | --- | --- | --- | --- |
| SNP (Coded Allele) | **Gene** | | **OR (95% CI)** | **p value** | | **OR (95% CI)** | **p value** |
| rs2736100 (T)* | *TERT* | | 0.67 (0.40; 1.11) | 0.115 | |  |  |
| rs2076295 (G)* | *DSP* | | 1.50 (0.93; 2.43) | 0.099 | | 1.14 (0.88; 1.47) | 0.303 |
| rs4727443 (A)* | *AZGP1* | | 0.66 (0.41; 1.06) | 0.088 | |  |  |
| rs7934606 (A)* | *MUC2* | | 0.86 (0.53; 1.38) | 0.522 | | 0.97 (0.78; 1.20) | 0.782 |
| rs2034650 (C)* | *IVD* | | 0.86 (0.54; 1.36) | 0.511 | |  |  |
| rs1981997 (A)* | *MAPT* | | 0.72 (0.39; 1.31) | 0.281 | | 0.89 (0.65; 1.21) | 0.448 |
| rs12610495 (G)* | *DPP9* | | 1.31 (0.76; 2.27) | 0.330 | |  |  |
| rs6793295 (C)* | *LRRC34* | | 0.67 (0.37; 1.21) | 0.183 | | 1.04 (0.79; 1.39) | 0.748 |
| rs2609255 (G)* | *FAMI3A* | | 0.78 (0.47; 1.29) | 0.325 | |  |  |
| rs11191865 (G)* | *OBFC1* | | 1.15 (0.72; 1.84) | 0.558 | | 0.97 (0.75; 1.25) | 0.834 |
| rs1278769 (A)* | *ATP11A* | | 1.22 (0.68; 1.19) | 0.511 | |  |  |
| rs1379326 (G)† | *CSMD1* | | 0.46 (0.13; 1.60) | 0.222 | | 0.65 (0.35; 1.22) | 0.181 |
| rs17690703(T)* | ***SPPL2C*** | | **0.61 (0.39; 0.96)** | **0.034** | | 0.86 (0.64; 1.15) | 0.314 |
